# Multislice CT of the head and body routine scans: Are scanning protocols adjusted for paediatric patients?

**DOI:** 10.2349/biij.8.1.e3

**Published:** 2012-01-01

**Authors:** Z Sun, KS Al Ghamdi, IH Baroum

**Affiliations:** 1 Discipline of Medical Imaging, Department of Imaging and Applied Physics, Curtin University, Perth, Australia; 2 Department of Radiology, King Abdul Aziz Hospital and Oncology Centre, Jeddah, Saudi Arabia

**Keywords:** Multislice CT, tube current, radiation, radiation risk, paediatric imaging

## Abstract

**Purpose:**

To investigate whether the multislice CT scanning protocols of head, chest and abdomen are adjusted according to patient’s age in paediatric patients.

**Materials and Methods:**

Multislice CT examination records of paediatric patients undergoing head, chest and abdomen scans from three public hospitals during a one-year period were retrospectively reviewed. Patients were categorised into the following age groups: under 4 years, 5–8 years, 9–12 years and 13–16 years, while the tube current was classified into the following ranges: < 49 mA, 50–99 mA, 100–149 mA, 150–199 mA, > 200 mA and unknown.

**Results:**

A total of 4998 patient records, comprising a combination of head, chest and abdomen CT scans, were assessed, with head CT scans representing nearly half of the total scans. Age-based adjusted CT protocols were observed in most of the scans with higher tube current setting being used with increasing age. However, a high tube current (150–199 mA) was still used in younger patients (0–8 years) undergoing head CT scans. In one hospital, CT protocols remained constant across all age groups, indicating potential overexposure to the patients.

**Conclusion:**

This analysis shows that paediatric CT scans are adjusted according to the patient’s age in most of the routine CT examinations. This indicates increased awareness regarding radiation risks associated with CT. However, high tube current settings are still used in younger patient groups, thus, optimisation of paediatric CT protocols and implementation of current guidelines, such as age-and weight-based scanning, should be recommended in daily practice.

## INTRODUCTION

The use of helical CT, in particular, multislice CT (MSCT), is rapidly growing due to technological improvements in modern CT scanners. Advances in CT imaging have resulted in a significant increase in the frequency of CT examinations in children. The number of CT examinations has been increasingly performed in the paediatric population in the United States and European countries [1–3]. The growth of CT use in children is mainly due to the decrease in the time needed to complete a scan, which is currently less than 1 second, largely eliminating the need for sedation or anaesthesia to prevent the child from moving during image acquisition. Although MSCT provides excellent opportunities for imaging children, scanning techniques have become more complicated due to great variability in body size in the paediatric population, and radiologists are faced with challenges of tailoring the scanning protocols to the small-sized paediatric patients.

Increasing use of CT becomes more problematic if the ALARA (as low as reasonably achievable) principle is not followed and CT techniques are not adjusted according to the age and size of the child [4, 5]. Although it offers improved diagnostic image quality, MSCT contributes more radiation dose than single-slice CT scans for various body regions. Recently, the practice of paediatric CT has been under increasing scrutiny due to the linkage between cancer and levels of childhood radiation [6]. Efforts to reduce and minimise radiation dose associated with paediatric CT have been made with significant progress since articles appeared in the February issue of 2001 American Journal of Roentgenology [6–8]. These articles discussed the potential risks associated with paediatric CT imaging, and highlighted a lack of attention to the radiation risks in children by paediatric CT protocols within the radiology community, while proposing suggestions or recommendations for adjusting CT technical parameters to minimise radiation dose. It has been reported that there is a greater use of age-adjusted body CT examinations [9], however, CT radiation awareness in paediatric imaging continues to be an important topic that should be given attention by both radiologists and clinicians.

According to the National Conference on Dose Reduction held in 2002, approximately 43% of imaging departments reported that they had introduced programmes to adjust CT parameters for children [10]. Although there is still adequate room for improvement, the change signals a dramatic and positive direction compared with the near-universal lack of such practices as early as 2001 [8].

CT dose reduction in paediatric imaging requires a combination of different approaches or strategies. These include optimisation of scanning protocols for children according to age- or weight-based adjustments, justification of paediatric CT use in paediatric clinics and emergency departments, decrease of unnecessary examinations, development of automatic exposure control devices by manufacturers, and user education for paediatricians and radiological technologists. Of these approaches, tube current is one of the key factors that must be modified as patients’ sizes vary widely. Adjustments of CT scanning protocols based on age and weight have been reported to be convenient and effective in clinical practice, according to early studies [11–13]. However, to our knowledge, very few reports are available in Saudi Arabia with regard to the investigation of paediatric CT scanning protocols. There are no standardised procedures for paediatric CT imaging across hospitals in Saudi Arabia, as each hospital has its own specific procedures, which are not necessarily optimised in terms of dose reduction.

The purpose of this study was to assess the paediatric CT practice, analyse CT scanning parameters used in routine head, chest and abdomen imaging, and investigate whether the CT protocols are appropriately adjusted according to the age of the children in major hospitals in Jeddah, Saudi Arabia. It is expected that the study results could be used by radiologists and medical imaging technologists to modify their existing practice and serve as a basis for optimisation of paediatric CT imaging.

## MATERIALS AND METHODS

The study population consisted of all paediatric patients seen at three major public hospitals in Jeddah, Saudi Arabia from May 2009 to May 2010. MSCT examination records were retrospectively reviewed for paediatric patients undergoing head, chest and abdomen MSCT scans during this one-year period. Inclusion criteria included patients 16 years old or younger; routine head CT and head trauma scans; routine chest CT; routine abdominal CT and abdominal trauma scans. For each CT examination, information was obtained and recorded from the CT scans which included the patient’s age, body regions examined and scanning parameters for each body region. These parameters included tube current (mA), tube kilovoltage and slice thickness. Since tube current is the main parameter that is commonly adjusted based on the patient’s age or body weight, it is characterised into the following ranges: < 49 mA, 50–99 mA, 100–149 mA, 150–199 mA, > 200 mA and unknown.

All CT scans were performed with 64-slice CT scanners in these three hospitals. Siemens 64-slice CT scanners (Somatom Sensation 64, Siemens, Forchheim, Germany) were used in hospitals A and B, while GE 64-slice scanner (GE Medical Systems, Lightspeed VCT, GE Healthcare, Milwaukee, USA) was used in hospital C. The scanning parameters for these CT scanners were variable, depending on the age of the patients, and details are summarised in Table 1. Tube voltage was 120 kVp, tube current was adjusted according to patient’s age and gantry rotation time was 330 ms. A spiral CT scanning mode was used on both types of CT scanners for acquisition of better images with minimal artefacts. For routine and trauma paediatric CT protocols, the slice thickness was 4.8–5.0 mm, 3 mm and 5 mm corresponding to the head, chest and abdomen CT, respectively.

**Table 1:** Tube current used for pediatric head routine CT in three hospitals.

**Tube current (mA)**	**Age group 0–4 years old**			**Age group 5–8 years old**			**Age group 9–12 years old**			**Age group 13–16 years old**		
	**Hospital A (No.)**	**Hospital B (No.)**	**Hospital C (No.)**	**Hospital A (No.)**	**Hospital B (No.)**	**Hospital C (No.)**	**Hospital A (No.)**	**Hospital B (No.)**	**Hospital C (No.)**	**Hospital A (No.)**	**Hospital B (No.)**	**Hospital C (No.)**
<49	0	0	0	0	0	0	0	0	0	0	0	0
50–99	0	0	18	0	0	24	0	0	11	0	0	0
100–149	196	0	0	0	0	0	0	0	0	0	0	0
150–199	0	277	0	67	89	0	0	0	0	0	0	17
>200	0	0	0	0	0	0	243	280	0	112	0	31
Unknown	0	0	0	0	0	0	0	0	0	0	367	0

No. Number of CT scans

Based on the categories by different ages, the patients were categorised into the following age groups: less than 4 years, 5–8 years, 9–12 years and 13–16 years. The patient’s weight factor could not be analysed as this information was not available.

Ethical approval was waived for this study, since only the CT scanning parameters were used for data analysis and no patient’s details were disclosed.

## RESULTS

**Figure 1:**
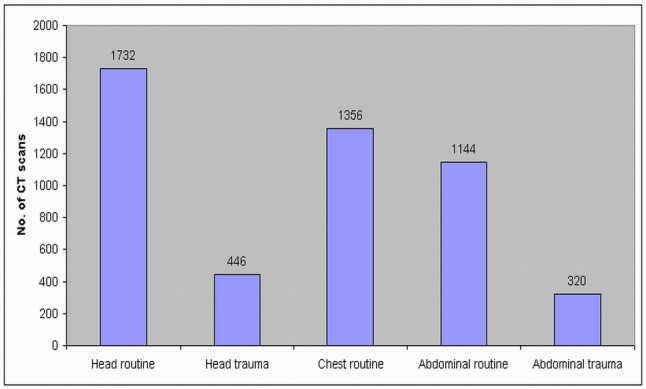
Distribution of the number of CT scans corresponding to different anatomic regions among the three hospitals.

A total of 4998 patient records comprising a combination of head, chest and abdomen CT scans among the three hospitals were assessed. 2178 out of 4998 scans were head CT examinations, which represented nearly half of all the CT scans (43.5%). The number of abdominal and chest CT scans was 1464 and 1356, respectively. Figure 1 shows the distribution of CT scans corresponding to the anatomic regions at these three hospitals.

Tables 2–5 show the number of CT scans with use of different mA ranges performed at each hospital corresponding to different age groups and anatomic locations. As shown in the tables, age-based adjusted CT protocols were used in most of the scans. The trend is to use a higher tube current setting with increasing age and slightly higher current setting for head and abdominal CT than for chest CT.

**Table 2:** Tube current used for pediatric head trauma CT in three hospitals.

**Tube current (mA)**	**Age group 0–4 years old**			**Age group 5–8 years old**			**Age group 9–12 years old**			**Age group 13–16 years old**		
	**Hospital A (No.)**	**Hospital B (No.)**	**Hospital C (No.)**	**Hospital A (No.)**	**Hospital B (No.)**	**Hospital C (No.)**	**Hospital A (No.)**	**Hospital B (No.)**	**Hospital C (No.)**	**Hospital A (No.)**	**Hospital B (No.)**	**Hospital C (No.)**
<49	0	0	0	0	0	0	0	0	0	0	0	0
50–99	0	0	3	0	0	6	0	0	10	0	0	0
100–149	8	0	0	0	0	0	0	0	0	0	0	0
150–199	0	50	0	12	28	0	37	0	0	0	0	0
>200	0	0	0	0	0	0	81	35	0	60	102	14
Unknown	0	0	0	0	0	0	0	0	0	0	0	0

No. Number of CT scans

**Table 3:** Tube current used for pediatric chest CT in three hospitals.

**Tube current (mA)**	**Age group 0–4 years old**			**Age group 5–8 years old**			**Age group 9–12 years old**			**Age group 13–16 years old**		
	**Hospital A (No.)**	**Hospital B (No.)**	**Hospital C (No.)**	**Hospital A (No.)**	**Hospital B (No.)**	**Hospital C (No.)**	**Hospital A (No.)**	**Hospital B (No.)**	**Hospital C (No.)**	**Hospital A (No.)**	**Hospital B (No.)**	**Hospital C (No.)**
<49	18	0	0	0	0	0	0	0	0	0	0	0
50–99	91	300	0	0	0	0	0	0	0	0	0	0
100–149	0	0	10	38	56	9	0	0	7	0	0	0
150–199	0	0	0	0	0	0	202	274	0	90	0	0
>200	0	0	0	0	0	0	0	0	0	126	118	17
Unknown	0	0	0	0	0	0	0	0	0	0	0	0

No. Number of CT scans

**Table 4:** Tube current used for pediatric abdomen routine CT in three hospitals.

**Tube current (mA)**	**Age group 0–4 years old**			**Age group 5–8 years old**			**Age group 9–12 years old**			**Age group 13–16 years old**		
	**Hospital A (No.)**	**Hospital B (No.)**	**Hospital C (No.)**	**Hospital A (No.)**	**Hospital B (No.)**	**Hospital C (No.)**	**Hospital A (No.)**	**Hospital B (No.)**	**Hospital C (No.)**	**Hospital A (No.)**	**Hospital B (No.)**	**Hospital C (No.)**
<49	0	0	0	0	0	0	0	0	0	0	0	0
50–99	67	153	0	0	0	0	0	0	0	0	0	0
100–149	0	0	0	78	47	0	0	0	0	0	0	0
150–199	0	0	2	0	0	6	150	111	9	0	0	0
>200	0	0	0	0	0	0	0	0	0	187	313	21
Unknown	0	0	0	0	0	0	0	0	0	0	0	0

No. Number of CT scans

**Table 5:** Tube current used for pediatric abdomen trauma CT in three hospitals.

**Tube current (mA)**	**Age group 0–4 years old**			**Age group 5–8 years old**			**Age group 9–12 years old**			**Age group 13–16 years old**		
	**Hospital A (No.)**	**Hospital B (No.)**	**Hospital C (No.)**	**Hospital A (No.)**	**Hospital B (No.)**	**Hospital C (No.)**	**Hospital A (No.)**	**Hospital B (No.)**	**Hospital C (No.)**	**Hospital A (No.)**	**Hospital B (No.)**	**Hospital C (No.)**
<49	0	0	0	0	0	0	0	0	0	0	0	0
50–99	12	7	0	0	0	0	0	0	0	0	0	0
100–149	0	0	0	36	19	0	0	0	0	0	0	0
150–199	0	0	2	0	0	4	47	28	2	0	0	0
>200	0	0	0	0	0	0	0	0	0	89	61	13
Unknown	0	0	0	0	0	0	0	0	0	0	0	0

No. Number of CT scans

In children 0–4 years old, a tube current of less than 50 mA was only used in 16% (18/109) of routine chest CTs in hospital A. A tube current of 150–199 mA was used in 7% of all CT scans for this age group, and this was mainly observed for the head CT scans in hospital B (50%), while in hospital A, there is no record of using more than 150 mA. None of the hospitals used more than 200 mA for these CT scans.

In children 5–8 years old, the most commonly used tube currents were between 100–149 mA and 150–199 mA. A tube current of 50–99 mA was used in 13% of all CT scans in Hospital C, and this was only observed in the head CT (both routine and trauma CT) scans representing 23% of all head CT scans.

When scanning the children 9–12 years old, increased tube current was used in most of the CT scans in Hospital A and B, while in Hospital C, a low tube current was still applied. A tube current of more than 150 mA was used in all of the CT scans in both Hospital A and B, representing 37% and 27% of all the CT scans. A tube current of more than 200 mA was used in more than 90% of the head CT scans in both hospitals. In contrast, a tube current of 50–99 mA was used in all of the head CT scans, and tube currents of 100–149 mA and 150–199 mA were used in chest and abdomen CT scans in hospital C. None of the CT scans used more than 200 mA in this age group in Hospital C.

**Figure 2:**
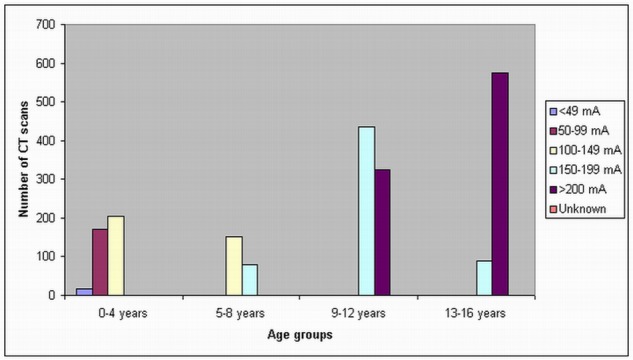
Tube current settings used for paediatric head, chest and abdominal CT scans in hospital A. High tube current is used with increasing age of patients, with a tube current of more than 200 mA being applied in patients older than 9 years old.

**Figure 3:**
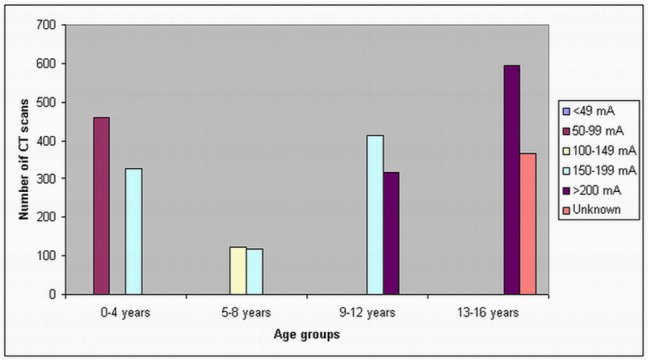
Tube current settings used for paediatric head, chest and abdominal CT scans in hospital B. A high tube current of 150–199 mA was used in the age group of 0–4 years old, although a tube current of more than 200 mA was only applied in the patients older than 9 years old.

**Figure 4:**
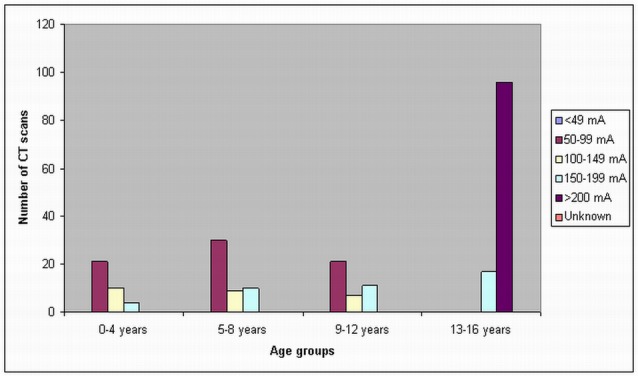
Tube current settings used for paediatric head, chest and abdominal CT scans in hospital C. High tube current is used with increasing age of patients, with a tube current of more than 200 mA being applied only in patients older than 13 years old.

**Figure 5:**
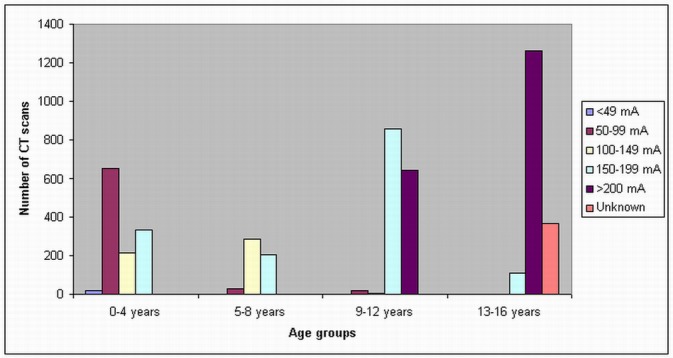
Tube current settings used for paediatric head, chest and abdominal CT scans among three hospitals. High tube current is used with increasing age of patients, however, a tube current of 150–199 mA was still used in a number of CT scans in the patients less than 5 years old.

In the children 13–16 years old, a tube current of 200 mA was used in most of the CT scans in all of these three hospitals, although a tube current of 150–199 mA was used in 55% of routine head CTs in Hospital C. In Hospital B, there is an unknown tube current of routine head CT in 367 scans. A scan delay of 25–30 sec and 50–60 sec was used in the chest and abdomen CT scans, while in head CT scans, the scan delay was shown to be dependent on the patients. Figures 2–4 show the number of CT scans in each age group corresponding to different tube current ranges in hospital A, B and C, while figure 5 is the distribution of CT scans in each age group among all of the three hospitals.

## DISCUSSION

This study was designed to investigate the current practice of MSCT for routine head and body scans in paediatric patients among three public hospitals in Saudi Arabia. There are three major findings arising from this study, which are considered valuable from a clinical perspective. First, the tube current of routine paediatric CT scanning protocols is adjusted according to patient’s age in most situations, indicating the awareness of dose reduction in imaging paediatric patients. Second, variable scanning protocols are used in different age groups, in particular, a high tube current is applied in paediatric patients from younger age groups. This reflects the fact that reduction in radiation dose and radiation protection needs to be increased in clinical practice, especially when scanning paediatric patients. Third, patients might be exposed to high radiation dose due to use of fixed scan delay in the contrast-enhanced CT scans, thus, optimisation of paediatric CT protocols is necessary in these hospitals.

Radiation dose in children from CT has increased significantly since the imaging modality has progressed from single-slice to helical CT and multislice CT examinations that are widely available today. Children are at greater risk than adults from a given dose of radiation, because they are inherently more radiosensitive to radiation exposure due to the increased number of dividing cells in growing children and the higher remaining years of life ahead of them, which indicates that they have more time to develop a radiation-induced cancer [11]. It is estimated that children are 10 times more sensitive to the effects of radiation than middle-aged adults [12, 13]. The risk of developing a radiation-induced cancer has been estimated to be 5% per Sv at all ages [14], however, this figure is close to 15% if the exposure occurs in the first decade of life [15]. According to the recent Biological Effects of Ionising Radiation (BIER VII) report [16], it is estimated that an exposure of 10 mSv carries a 1 in 1000 risk of developing a solid cancer or leukaemia.

Brenner *et al.* [11] assessed the lifetime risks of developing a fatal cancer attributable to radiation from two common routine paediatric CT examinations, namely abdominal and head examinations. Their estimates suggested that the risk of dying from cancer is approximately 1 in 550 for a single abdominal CT examination and 1 in 1500 for a head CT examination if the scan is performed in a 1-year-old child. However, it is necessary to point out that these estimates were based on the assumption that the same CT scanning protocols used in adult examinations were applied in children without adjustments. Thus, the risk would be lower if paediatric CT protocols were adopted and the paediatricians were aware of applying specific protocols in imaging children.

If other parameters (tube voltage, scan time, filtration and section thickness) are fixed, as is often the case, tube current is the primary factor in determining radiation dose to patients, and it should be modified not only with reference to patient’s age but also to their weight as patient’s sizes vary widely. Different approaches can be used to optimise the tube current settings [2, 3, 17–19]. Adjustments of CT scanning protocols based on weight and age are found to be convenient approaches for dose reduction in clinical practice, according to early studies [17, 18]. For head CT scans, tube current should be modified according to different age groups because the attenuation in the head largely depends on the thickness of the skull, which changes with age [18]. Suess and Chen suggested that after the age of 6 years, adult tube current settings can be used since the size of the head and the ossification of the skull would have almost reached the adult levels [18]. The results from this study showed that age-based scanning is practised in two out of three hospitals, while in another hospital (hospital B), a tube current of more than 150 mA was still used for both routine and trauma head CT scans in children between 0 and 4 years old. A tube current of more than 150 mA was used in the age group of 9–12 years for head, chest and abdomen CT scans, and more than 200 mA for all of these body regions in children more than 13 years old. This is much higher than the recommended ranges of 91–130 mA for cranial and 76–90 mA for thoracic, abdominal and pelvic CT examinations by Shah *et al*. [20]. Thus, optimisation of these paediatric CT protocols is necessary for dose reduction.

For paediatric body CT (chest/abdomen/pelvis) protocols, modification of scanning protocols was suggested to be made based on weight [2, 7, 17]. Although information regarding body weight was not available in this study, the tube current was adjusted based on patient’s age among chest and abdominal CT scans in hospitals A and B, while in hospital C, a consistent tube current was used across all age groups. This indicates the possibility of exposure of the paediatric patients to higher radiation dose.

Adjustment of tube current is not only based on the age or the weight of patients, it is also controlled by using automatic current modulation techniques to reduce radiation dose in paediatric CT examination without affecting diagnostic image quality [21]. Patient’s body attenuation is measured online instead of manually performed during the scan, and the tube output is controlled for all viewing angles according to the detected attenuation. This helps to reduce radiation exposure in all types of patients and body regions. Clinical studies have confirmed very efficient dose reduction based on online tube current modulation [22–26]. Greess *et al.* in their studies concluded that a significant dose reduction was achieved in the thorax and abdomen with use of attenuation-based online modulation, resulting in up to 20 to 40% dose reduction without compromising image quality [27, 28]. This feature has been implemented in many modern CT scanners, thus, it has the potential to work as an automatic exposure control for paediatric dose reduction when compared to conventional exposure control methods. Manual adjustments of tube current in paediatric CT scans were conducted among the three hospitals in this study, and this may explain the variable tube current ranges used in different CT scans. More importantly, the online modulation of tube current allows for acquisition of desired noise level in different anatomical regions or in patients of different sizes [18]. Thus, automatic exposure control is recommended in paediatric CT imaging with use of modern CT scanners. Peng *et al.* compared the study group using automatic tube current modulation (mAs ranging from 20–79 mAs) with the control group using fixed mAs (120 and 150 mAs) [27]. A reduction of 65% radiation exposure was achieved in the study group, while the image quality was clinically acceptable, despite the increased image noise measured with lower mAs settings.

Peak kilovoltage (kVp) is another key factor that determines radiation dose in CT imaging. Smaller volumes are scanned in paediatric CT imaging, so tube voltage should be reduced accordingly. A standard 120 kVp setting for adult CT protocols is no longer suitable for paediatric imaging, especially in young patients as the size and weight distribution in paediatric patients is different from adults. However, this was not observed in this study, as 120 kVp was consistently used across all age groups of paediatric CT scans. This should be given attention by radiologists/radiographers when setting up the scanning protocols. 80 kVp or 100 kVp setting has been widely adopted for paediatric CT imaging with satisfactory diagnostic images achieved [2, 28, 29]. Lowering of the tube voltage to 80 kVp in children was recently recommended in paediatric CT without compromising image quality [29–31]. Lee *et al.* in their study showed that the average dose length product in children with congenital heart disease was reduced by 70% at 80 kVp when compared to that acquired at 120 kVp [30]. Saad *et al.* combined a tube voltage of 80 kVp and adjusted tube current using dual-source CT angiography in 110 infants with congenital heart disease, and their results demonstrated a significant reduction of radiation dose without impairing image quality [28]. Therefore, a combination of adjusted kVp and tube current is highly recommended for further dose reduction to paediatric patients undergoing CT examinations.

Optimised contrast medium administration is essential for the visualisation of anatomical structures, detection of pathological changes and assessment of disease extent. There are three different bolus timing techniques for CT contrast enhancement: fixed scan delay, scan delay estimation from a test-bolus injection, and real-time bolus-tracking techniques [32, 33]. Since there is a large variation between circulation times of patients with cardiovascular diseases, fixed scan delay is no longer practical for contrast-enhanced CT. Scan delay can be individualised by using a test bolus or a bolus tracking technique. A fixed scan delay was used in chest and abdomen CT scans in these hospitals, thus it is possible that patients are exposed to high radiation dose due to suboptimal contrast enhancement and longer exposure time.

Some limitations in this study should be addressed. Firstly, although routine head, chest and abdominal CT scans were included in this study, the analysis was only based on scans of individual body region. Clinically, it is possible that patients will undergo a CT scan covering multiple anatomic regions, such as combined chest and abdomen or abdomen-pelvis scans. Thus, this factor needs to be taken into consideration. Secondly, body weight of patients was not available for this analysis, which is another main limitation, as guidelines have been provided for size-based adjustments in paediatric examination parameters [7, 34]. Thirdly, this study did not address the issue of image quality and diagnostic value. It is important to lower radiation dose in paediatric CT imaging, but at the same time, it is essential to achieve quality diagnostic images which are acceptable in answering all clinical questions. Further research is needed to investigate the complex relationship between radiation exposure, image quality and diagnostic value of paediatric CT imaging to establish the minimum radiation dose necessary to provide information that is sufficient for clinical diagnosis.

## PRACTICAL RECOMMENDATIONS

It is important to reduce radiation dose but still maintain acceptable image quality in paediatric CT imaging. Efforts have taken place in recent years to increase awareness about adult and paediatric radiation protection, and Image Gently represents one of these campaigns [35–37]. The following suggestions are recommended to achieve the low-dose CT paediatric imaging:

- Perform CT examinations only for appropriate indications;
- Use published age-adjusted or weight-related parameters for CT in children;
- Manufacturers need to be more user-friendly and provide the tools to prevent excess doses, such as automatic exposure control. A fixed milliamperes protocol must be avoided;
- More research on dose reduction by reduction of kVp and maintenance of diagnostic image quality;
- Practitioners, including paediatricians and paediatric radiologists must follow the guidelines regarding referral criteria for paediatric CT imaging.

In conclusion, this analysis shows that paediatric CT scan parameters in the three surveyed hospitals are adjusted according to the patient’s age in most of the routine CT examinations. This indicates increased awareness about radiation risks associated with CT. However, variable scanning parameters are used in the much younger age groups i.e. less than 4 years old, potentially delivering higher radiation dose to this group. This emphasises the importance of implementing and adhering to appropriate guidelines in paediatric CT imaging such as age- or weight-based scanning in daily practice. Information on current practices in multislice CT in children should serve as a foundation for future recommendations and investigations into multislice CT in paediatric patients.
